# The SARIFA biomarker in the context of basic research of lipid-driven cancers

**DOI:** 10.1038/s41698-024-00662-2

**Published:** 2024-07-31

**Authors:** Bruno Märkl, Nic G. Reitsam, Przemyslaw Grochowski, Johanna Waidhauser, Bianca Grosser

**Affiliations:** 1 Pathology, Medical Faculty Augsburg, University of Augsburg, Augsburg, Germany; 2 Bavarian Cancer Research Center (BZKF), Augsburg, Germany; 3 WERA Comprehensive Cancer Center, Augsburg, Germany; 4 Hematology and Oncology, Medical Faculty Augsburg, University of Augsburg, Augsburg, Germany

**Keywords:** Prognostic markers, Cancer metabolism, Oncogenesis

## Abstract

SARIFA was very recently introduced as a histomorphological biomarker with strong prognostic power for colorectal, gastric, prostate, and pancreatic cancer. It is characterized by the direct contact between tumor cells and adipocytes due to a lack of stromal reaction. This can be easily evaluated on routinely available H&E-slides with high interobserver agreement. SARIFA also reflects a specific tumor biology driven by metabolic reprogramming. Tumor cells in SARIFA-positive tumors benefit from direct interaction with adipocytes as an external source of lipids. Numerous studies have shown that lipid metabolism is crucial in carcinogenesis and cancer progression. We found that the interaction between tumor cells and adipocytes was not triggered by obesity, as previously assumed. Instead, we believe that this is due to an immunological mechanism. Knowledge about lipid metabolism in cancer from basic experiments can be transferred to develop strategies targeting this reprogramed metabolism.

## Introduction

**Fig. 1:**
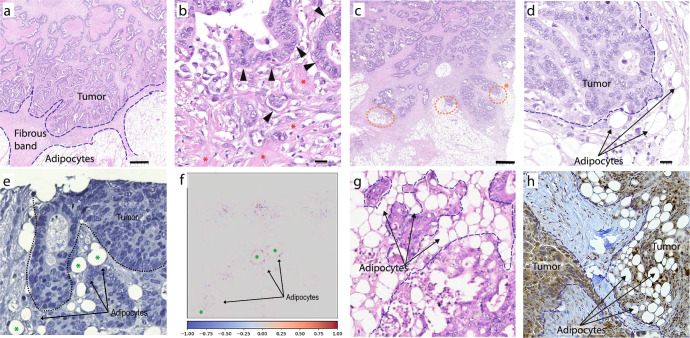
Histology examples of tumor-adipocyte interaction. **a** H&E, Scale bar: 500 µm, SARIFA-negative colon cancer. Tumor cells do not touch any adipocytes but are separated by a continuous fibrous band. **b** High magnification of a. Scale bar: 20 µm, Tumor glands (arrowheads) are surrounded by collagen fibers (red stars). **c** H&E, Scale bar: 500 µm, SARIFA-positive colon cancer. Tiny areas of fat get into contact with tumor glands (orange circles). **d** High magnification of c (corresponding to circle with star). Scale bar: 20 µm, Tumor cells come into direct contact with adipocytes, which decrease in size. **e** Courtesy of S. Foersch et al.^6^. Crop from an IHC staining from a study using a deep learning algorithm analyzing immune cells in colorectal cancer: adipocytes in proximity to tumor cells. **f** Heat map of prognostically relevant structures corresponding to **e**. This explainable artificial intelligence approach identified not only immune cells but also adipocytes (green stars in **e** and **f**) close to a tumor as prognostically relevant. **g** H&E, courtesy of E. Wulczyn^3^. The tumor adipose feature (TAF), identified by deep learning, has a significant overlap with SARIFA. **h** Courtesy of A. Mukherjee^88^. FABP4 immunohistochemistry; Ovarian cancer metastasized to the omentum show contact with shrinking adipocytes and strong expression of FABP4.

We recently introduced SARIFA as a prognostic biomarker in cancer. SARIFA, an acronym for Stroma AReactive Invasion Front Areas, describes the histomorphological phenomenon of direct contact between tumor cells and adipocytes without intervening collagenous or inflammatory stroma. In gastric and colorectal cancer, SARIFA positivity is present when at least five tumor cells with direct contact with adipocytes are identified^1,2^ (Fig. 1). In contrast, in pancreatic and prostate cancer, a quantitative cut-off has to be defined to achieve better prognostic discrimination. The threshold of five tumor cells has been chosen to define a clear distinction from tumor budding, defined as single cells and clusters of up to four tumor cells. Because the evaluation is based on conventional light microscopy on H&E-stained routine slides, this marker is characterized by unlimited and fast availability, as well as virtual no additional costs. Wulczyn et al. published a very similar but not identical feature named tumor adipose feature (TAF) analyzed by a deep learning approach in the same year as we reported SARIFA^3,4^. Briefly, the authors here aimed at predicting survival of CRC patients directly from routine H&E slides, then explored the human interpretability of important features and hereby identified TAF. In a subsequent pathologist validation study, they could prove that TAF as a machine learning-derived histopathologic feature could indeed be learned and scored by pathologists (3, 4). In similar approaches, Foersch et al. and Jiang et al. both also independently identified the proximity of adipocytes and tumor cells as an unfavorable factor in CRC by deploying DL-algorithms^5,6^.

**Fig. 2:**
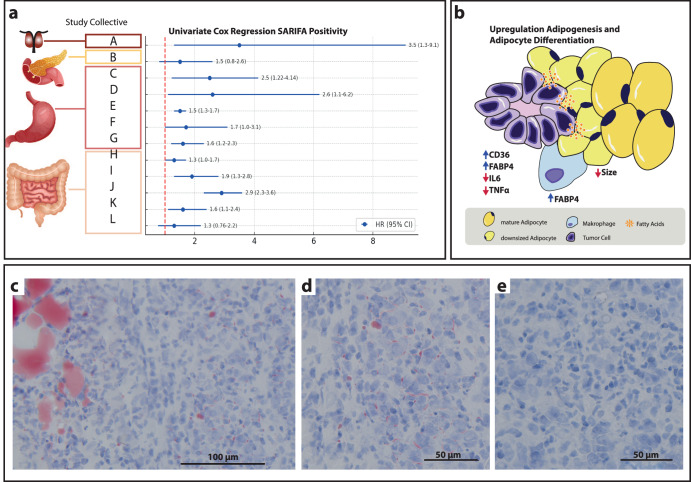
Prognostic effect and metabolic reprogramming of SARIFA-positive cancers. **a** Cox regression analyses in different cancer entities and cohorts: (A) overall survival (OS) – prostate cancer (Enke 2024); (B) OS – pancreatic cancer (Grochowski 2024); (C, D, E, F, G) OS – gastric cancer ST03 trial, OS – gastric cancer MAGIC trial, OS – gastric cancer TUM-cohort, OS – gastric cancer Augsburg validation set, OS – gastric cancer Augsburg test set (Grosser 2023 and 2022); (H, I, J) OS – colorectal Netherlands cohort study, progression-free survival – Düsseldorf colorectal cohort, OS – TCGA colorectal (Reitsam 2023 and 2024); (K, L) OS – colon cancer Augsburg validation set, and OS – colon cancer test set, respectively. **b** SARIFA and illustration of lipid-driven tumor biology. CD36 = cluster of differentiation 36 (fatty acid translocase), FABP4 fatty acid binding protein 4, IL6 interleukin 6, TNFα tumor necrosis factor alpha. **c** Red Oil staining. Scale bar: 100 µm. Poorly cohesive gastric cancer. Lipid within adipocytes is identified by red staining. **d** Higher magnification of *c*, scale bar: 50 µ, showing lipid within the cytoplasm of tumor cells. **e** Red Oil staining. Scale bar: 50 µ. Same cancer as *c* with an area from the tumor center. No lipids can be appreciated.

The distinct morphology of SARIFA-positive tumors suggests, from a sole histopathological point of view, a certain degree of ‘*defenselessness*’ of the host organism against the tumor cells and clusters, which can apparently penetrate almost unhindered through healthy tissue into deeper structures. In contrast, tumor growth in SARIFA-negative cases is accompanied by a histologically visible stromal reaction consisting of fibroblast proliferation and the production of collagen fibers (Fig. 2). However, an altered tumor-host response is likely only one aspect of this tumor biology, reflected by the SARIFA morphology. More importantly, it seems that the metabolic effects of the direct and intense interaction between tumor cells and adipocytes lead to a lipid-dominated metabolism of the tumor cells^1,7–10^. This distinct and direct interplay between tumor cells and adipocytes, including various components of the tumor microenvironment (TME), has already been the subject of clinical and intensive experimental research^11^. The fact that obese people are more frequently affected by cancer and have a more aggressive clinical course compared to lean people is the primary motivation for this intensive research^12,13^. There is overwhelming evidence that direct interaction between adipocytes and tumor cells promotes aggressive features by leading to a metabolic switch, serving as an energy source, and producing cellular structure elements^14,15^. Current anticancer therapy approaches include surgery, radiation, conventional chemotherapy, antihormonal therapy, induction of differentiation, antiangiogenic therapy, targeted therapy, and immune therapy. Targeting lipid metabolism might be a new approach to attacking cancers. Moreover, SARIFA could potentially predict its effectivity by indicating direct adipocyte tumor cell interaction in the future^16–18^.

As outlined in the following, SARIFA shows a broad overlap with many insights from the aforementioned experimental findings. However, up to now, our group has not identified an association between SARIFA and obesity. We, therefore, hypothesize that SARIFA is not driven by overweight but by other mechanisms that are very likely linked to immunology.

This review aims to summarize (i) the prognostic value of SARIFA and the translational findings of this phenomenon, (ii) the role of lipid metabolism in cancers in general, and (iii) the context of SARIFA as the main driver for its aggressiveness.

## Stroma AReactive Invasion Front Areas – SARIFA

### SARIFA as a prognostic marker

SARIFA classification was confirmed as prognostic in colorectal (*n* = 2547), gastric (*n* = 2495), pancreatic (*n* = 174), and prostate cancers (*n* = 301). Especially in gastric and colorectal cancers, a strong prognostic effect could be seen in several independent retrospective cohorts, partially from clinical trials. SARIFA positivity is statistically independently associated with progression-free and overall survival in digestive cancers, as well as several other adverse features^1,2,8,10,16,18^. Very recently, the first analysis of a different group has been published, confirming the independent prognostic effect of SARIFA in gastric cancer^19^. In prostate cancer, a correlation of SARIFA with other adverse factors such as extensive extraprostatic growth, number of affected lymph nodes, and higher Gleason score could be shown. The association with the occurrence of bone metastases and removed lymph node metastases just failed the significance level^9^. The SARIFA classification–related uni- and multivariate hazard ratios (SARIFA positive vs. SARIFA negative) for the various cancer entities are summarized in Fig. 2 and Supplementary Fig. 1. Importantly, the H&E-based histopathological evaluation that relies on assessing surgical resection specimens (Fig. 1 and Supplementary Figs. 2–5) is remarkably easy and fast. In colon cancer, the mean evaluation time per case analyzing one slide was only 21.6 s^2^. Therefore, even in a case with many tumor slides, the additional evaluation time will hardly exceed two minutes. No additional staining, immunohistochemistry, or advanced devices are necessary, resulting in virtual time and cost neutrality. SARIFA status is not influenced by typical demographic data, such as sex or age, or, importantly, by the body mass index (BMI) at diagnosis^1,2,7–10,16,18^. The latter seems to be especially relevant because the interplay between adipocytes and tumor cells might be facilitated by obesity, although this does not seem to be the case, according to our current knowledge. Naturally, SARIFA can only occur where tumor cells can get into contact with adipocytes where fat anatomically is located which means that SARIFA is predominantly found in locally advanced cases. However, it has to be kept in mind that adipocytes can be found in the submucosal layer in the GI tract. In concordance, SARIFA-positive cases have been identified in pT1/2 cases of gastric and colorectal cancers^1,7,8,16,18^.

The extent of SARIFA can differ considerably between different entities and between cases of the same entity. As a consequence, a quantitative cut-off for SARIFA positivity has to be established for pancreatic ductal cancer because areas of SARIFAs can be identified in most cases^10^. As mentioned above, gastric and colorectal cancers show SARIFA in only 16–53% of cases^1,2,8,16^, and SARIFA positivity is given from the moment it is identified, even if this is the case in only a single region of all available tumor-containing slides. Therefore, it is obviously necessary to investigate whether SARIFA has a quantitative effect on prognosis. We demonstrated this effect in a clinical trial cohort of gastric carcinomas in which the entire tumor material was embedded and evaluated with regard to SARIFA^20^. The prognostic effect of SARIFA also correlated with the proportion of SARIFA-positive sections, but only to a relatively small extent. Surprisingly, the most significant difference between the survival curves occurred between negative and initially positive cases^20^. An identical effect was shown by Wulczyn et al. for a phenomenon they named the tumor adipose feature (TAF)^3,4^, which shows considerable overlap with SARIFA and is summarized in one of the following paragraphs (‘SARIFA and Artificial Intelligence Technologies’).

Differences in the prognostic value between TAF absent versus present and absent versus widespread could be seen, but this was to a minor extent. We consider this a strong indication that SARFIA discriminates between two substantially different tumor biologies relying on the tumor-adipocyte interaction.

### SARIFA as a predictive marker

In addition to prognosis, the prediction of treatment response may be an even more important biomarker feature. No prospective studies have yet been performed, although they are necessary to adequately address the question of the predictive value of SARIFA evaluation. However, there have already been some clear hints from retrospective analyses of clinical trials in gastric cancer and the exploitation of TCGA RNA expression data in colorectal carcinomas^8,16,17^. Based on the results of the MAGIC trial, which compared surgery alone versus perioperative chemotherapy with epirubicin, cisplatin, and fluorouracil (ECF), one can conclude that only SARIFA-positive patients benefited from this chemotherapy regimen, although it is no longer the standard of care^16^. Gene expression-based drug response prediction revealed potentially reduced efficacy of oxaliplatin in SARIFA-positive colorectal cancers, although such cancers seemed to be more sensitive to dasatinib^8^.

### Association with lipid metabolism, FABP4, and CD36

Bulk as well as spatial RNA-expression analysis in gastric and colorectal cancer using our own cases and publicly accessible TCGA data^21–23^ revealed upregulation of lipid-associated genes in tumor cells and the TME of SARIFA-positive cancers. The top five upregulated genes in gastric cancer were all related to lipid metabolism. These findings provide a strong argument for the essential role of lipid metabolism in tumors characterized by direct contact with tumor cells and adipocytes. Especially striking is the upregulation of fatty acid binding protein (FABP) 4 in all tumor compartments, including the TME in SARIFA-positive tumors, on both RNA and protein levels. This important finding has been established in spatial RNA-expression analyses in gastric cancer^1,7^ and has been confirmed in bulk RNA analyses gastric cancer^7^ and colorectal cancer^8^. Immunohistochemically, FABP4 upregulation has been shown in gastric cancer^1^ and pancreatic cancer^10^. Within the TME, tumor-associated macrophages (TAMs) also show distinct upregulation of FABP4. As described in the following section, FABP4 is one of the most essential proteins responsible for lipid metabolism–associated tumor-promoting effects. CD36, a protein that realizes the uptake of FAs into the cells, has been shown to be elevated in gastric, pancreatic, and prostate cancer at the protein level (Fig. 2)^1,7–10^.

### Role of genetics

When exploiting the TCGA database and our own cases, there was no association identified between genetic aberrations and the SARIFA-status in either gastric or colorectal cancer. In colorectal cancer, this is especially true for typical driver mutations, such as the BRAF and KRAS genes, which are not enriched in SARIFA-positive CRCs. In line with this, no differences have been found regarding microsatellite status. Regarding the RNA expression-based molecular classification, CMS1 (MSI-immune) and CMS4 (mesenchymal) were found to be enriched among SARIFA-positive cases^8,24^. In concordance with this, genetic aberrations and TCGA molecular subtypes^21^ in gastric cancer have been seen to be randomly distributed within SARIFA-positive and negative cases^7^, indicating that SARIFA status is not influenced by genetics.

### Influence of the plasminogen system and immunology on SARIFA

Because typical mutations can be widely excluded as responsible for the occurrence of SARIFAs^7,8^, matrix degradation and immunoreaction could instead play an essential mechanistic role. Indeed, we found upregulation of the plasminogen system in SARIFA-positive colon cancers. Re-evaluating previously published studies^25,26^, the proteases urokinase plasminogen activator (uPA) and plasminogen activator inhibitor (PAI1) were found to be significantly elevated^27^. Both proteins are involved in matrix degradation in wound healing and neoplastic processes^28^. Because at least a partial lack of enclosing fibrous tissue is a major feature of SARIFA, it seems plausible that the upregulation of uPA/PAI1 has a causal function for SARIFA. Using RNA in situ hybridization, we investigated the cytokines interleukin (IL)6, IL10, IL12, and tumor necrosis factor (TNF)α in gastric cancer. While IL10 and IL12 showed no differences according to the SARIFA status, the proinflammatory cytokines IL6 and TNFα were downregulated in SARIFA-positive cases^7^. In colon cancer, a strong reduction in natural killer cells in peripheral blood and tumor tissue could be found in SARIFA-positive cases^29^. Although these hints need further confirmation to draw final conclusions, both studies showed a reduced immune-defensive reaction in SARIFA-positive cases, suggesting immunosuppressive status as an inhibitor of the local response against the tumor cells. An open question is whether this suggested reduced immune response plays a substantial role in SARIFA-related aggressivity in SARIFA development or even both.

### SARIFA and Artificial Intelligence Technologies

Simultaneous to our discovery of SARIFA on H&E light microscopy, Wulczyn et al. identified a very similar phenomenon through an artificial intelligence (AI) approach in colorectal cancer, which they named the tumor-adipose feature (TAF) (Fig. 1)^3,4,30^. In a recent collaborative study, the comparison of both morphological features revealed a broad, but not complete, overlap. Out of 175 available patches classified as TAF, 117 have been assigned to be SARIFAs. Interestingly, the increase in RNA expression of the lipid metabolism-related genes CD36 and FABP4 was stronger in SARIFApos/TAFpos cases compared to SARIFA-negative/TAF-positive cases. Classification of TAF did not reveal survival differences in SARIFA-negative cases^31^. This underlines the importance of direct contact between tumor cells and adipocytes, an imperative feature of SARIFA but not necessarily given in TAF. Both approaches confirm each other suggesting a new way of biomarker development combining analogous and digital attempts. Foersch et al. also established a deep-learning algorithm to predict prognosis and therapy response using an immune score-based multilayer approach. Unexpectedly, the generation of guided gradient-weighted class activation map (guided Grad-CAM) markup images revealed an adipocytes-close-to-tumor-cells feature as an immune reaction–an independent pattern that was prognostically relevant (Fig. 1)^6^. Jiang et al. came to similar conclusions in their recently published multicenter end-to-end prognosis study, once again highlighting the importance of tumor-infiltrated fat/tumor–adipocyte interaction for poor prognosis (see their manuscript in Supplementary Fig. 14)^5^. Therefore, the feature of tumor–adipocyte interaction has been identified in parallel and independently by conventional microscopy and by information technology using AI, underlining the significance of this biomarker.

## Lipid metabolism in cancer

While little attention has been paid to this topic in the past, metabolic reprogramming has now been included in the hallmarks of cancer, and adipocytes have been recognized as relevant to neoplastic processes due to their potential to fuel tumor cells^32,33^. Nevertheless, as early as the 1960s, fundamental research discovered an enhanced need for lipids, including in activated lipid uptake and synthesis^34–36^. There is growing evidence that lipids play a critical role in cancer biology, and subsequently, in cancer progression. Notably, this is not restricted to one or a few entities, but it rather appears to be a general principle^15^. In addition to de novo lipogenesis, cancer cells can uptake exogenous lipids, especially in the form of fatty acids (FAs). The following section will touch upon the main topics of lipid metabolism in cancer, providing a general understanding of the subject. However, a thorough explanation would exceed the scope of this review article. There are several outstanding overviews available that delve deeper into each individual topic and are cited below, for reference.

### Energy resource

Otto Warburg developed the concept of aerobic glycolysis as a preferential metabolic energy–providing mechanism^37,38^. Since its introduction, this concept has become essential to our understanding of cancer biology. In addition, there is fundamental knowledge that ATP production by FA-β-oxidation (FAO) plays an important role in tumor cells^39–42^. FAO appears to be especially enhanced if tumor cells come into direct contact with adipocytes^14,43^. Carracedo et al. summarized the particular importance of FAO in cells under the condition of loss of intercellular adherence with inhibition of glucose uptake^44^. They also stressed that aerobic glycolysis and upregulation of lipid metabolism are key interdependent metabolic processes in cancer progression. Both the Warburg effect and lipid metabolism can be attacked by a small molecule^45^. Interestingly, there are data supporting the particular importance of FAO for metastases formation. Detached melanoma cells upregulate FAO to supply their demand for NADPH. Knockdown of the FAO essential proteins TPβ and NUR77 reduces circulating tumor cells^46,47^. On the other hand, NUR77 inhibits the uptake of FAs and consecutive proliferation^48^. However, the latter is of minor importance during the initial tumor cell dissemination and distant colonization phase.

### Effects on cancer immunology

The tumor microenvironment (TME) reflects the interface between the tumor and the affected organism and hosts the defensive elements of the innate and adaptive immune systems. Lipids can modify the function of many kinds of immune cells, and they are, therefore, of crucial importance^15^. They can foster the function of immunosuppressive myeloid-derived stem cells (MDSCs), tumor-associated macrophages (TAMs), T-helper 2 (Th2) cells, Treg cells (Tregs), and dendric cells (DCs) as well as tumor-associated neutrophils (TANs)^49–54^. MDSCs within a lipid-rich tumor environment switch from glycolysis to FAO to cover their energy requirements^55^, generating a competitive advantage over antitumor immune cells^56^. Blocking FAO in mouse models led to a decreased immunosuppressive function^49^. An increased supply of FAs fosters the generations of protumoral TAMs with M2-phenotype with an immunosuppressive function due to activated JAK1-STAT6 signaling^57,58^. Tregs also benefit from CD36-mediated FA intake^51^. DCs are a major component of the MHC-II-class antigen-presenting system and show an impaired function by an abnormal lipid intake^59,60^. The cytotoxic features of CD8 T cells are impaired in cholesterol-rich TME, which is associated with increased CD36 expression and poor survival^61^. Prostaglandin E2 inhibits the release of DC attracting chemokines by natural killer (NK) cells^62^. FAO facilitates the responses against infection and cancer of NK cells with the help of CPT1A expression^63^. On the other hand, CPT1A increases the resistance of tumor cells against the cytotoxic function of T-cells^64^. In summary, lipids can effectively inhibit the immunologic system and contribute to cancer progression.

### Specific FA-transporting proteins

Several proteins are involved in the trans- and intracellular transport of FAs. Together with CD36, the family of FABPs has been investigated most frequently in the context of cancer.

#### CD36

CD36 (alternatively, platelet glycoprotein 4; fatty acid translocase, FAT; or scavenger receptor class B member 3, SCARB3) is a membrane protein that is responsible for the uptake of FAs into the cell and is therefore, in addition to de-novo lipogenesis, of crucial importance for the supply of lipids to the cells from the outside in. Very recently, Xia et al. provided an excellent and comprehensive review of this topic^65^. The upregulation and prognostic relevance of CD36 in cancers have been reported in several entities, such as gastric cancer, colorectal cancer, ovarian cancer, prostate cancer, clear cell renal cancer, and squamous cancer^66–72^. The extent of the differential expression and prognostic effect, however, seems to be entity dependent^73^. In addition to lipid-related ligands, CD36 has many other ligands, as underlined by Xia et al. Except for thrombospondin (TSP), most show a tumor-promoting effect^65^. Of interest are data suggesting CD36’s crucial role in metastatic homing. Driven by cancer-associated adipocytes (CAAs), the upregulation of CD36 in tumor cells facilitates the uptake of FAs in the omental metastasis of ovarian cancer. This mechanism is believed to contribute to the strong preference to metastasize into the neighboring omental fat^70,74^. For the possible therapeutic exploitation of CD36 as a target protein, see below.

#### FABP4

FABPs bind to long-chain FAs and accomplish intracellular traffic. The role of the isoform-comprising family in cancer has been comprehensively summarized by McKillop and colleagues^75^. Of the 10 members of this family, FABP4 is most frequently addressed in the context of cancer biology and therapy. Physiologically, FABP4 is expressed on the protein level mainly in white and brown adipocytic tissue. The human protein atlas additionally mentions respiratory tract and female tissues as regions of physiological protein expression^76,77^. It binds and transports saturated and unsaturated lipids as well as FAs. FABP4 is, together with CD36, responsible for the transmembrane lipid uptake^76^. Differential and aberrant gene expression of FABP4 is found in several cancer entities, such as breast cancer, non-small lung cancer, prostate cancer, colorectal and gastric cancer, and is associated with more aggressive clinical courses compared to low-level cases^78–85^. Of interest, FABP4 expression in ovarian tumor cells is restricted to metastases in which direct contact with adipocytes is realized^86,87^, emphasizing the importance of a lipid-rich environment and lipd-metabolism-associated proteins such as CD36. Cell culture and animal experiments in ovarian cancer revealed upregulation of lipid-metabolism proteins. FABP4 has been identified as being of particular importance. Knockdown- and knockout experiments led to reduced metastatic burden. The same was true after implication of a FABP4-inhibitor^88^. Regarding the possible therapeutic exploitation of FABP4 as a target protein, see below.

### Cancer-associated adipocytes

Based on localization and function, adipose tissue is divided into white and brown adipose tissue (WAT and BAT, respectively). BAT serves as a thermoregulator and is localized in the paracervical and supraclavicular regions. Wholly underestimated in its importance in the past, WAT stores energy and is a source of hundreds of hormones and functional proteins. It is localized in the subcutaneous region and around visceral organs^89^. Mature adipocytes interact with tumor cells when they come into direct contact with each other. This is the case if the regional anatomy of a tumor contains fat, which is especially the case in breast, gastrointestinal, pancreatic, and urothelial cancers and melanoma. Moreover, fat also occurs abundantly in some metastatic sites, such as the peritoneum and bone marrow. Mature adipocytes become delipidated and lose size, which is visible by light microscopy, and obviously provide lipids, including FAs, after lipolysis^10^. Moreover, adipocytes undergo a fundamental change in terms of phenotype and secretory function. In particular, proteinases such as MMP-11 and PAI-1 and inflammatory cytokines such as TNFα, IL-6, IL-1β are upregulated and secreted more strongly. Furthermore, adipokines are regulated toward a tumor-promoting function^90,91^. Finally, mature adipocytes become cancer-associated adipocytes (CAA) and can even transform into fibroblasts^89,92^. Co-culturing tumor cells and adipocytes leads to a significantly increased aggressiveness of tumor cells, as shown in animal experiments where tumor cells have been injected into the tails of mice^90^. This finding seems especially interesting because it supports the concept of a promoting effect that is not restricted to local tumor growth but also to dissemination and distant implantation. Importantly, cellular interaction is bidirectional and includes the regulatory influence of adipocytes through attached tumor cells. The activation of lipolysis within adipocytes induced by tumor cells is only one example of a wide range of reactions^87,93–95^.

### Lipid-induced anticancer therapy resistance

There is a large body of evidence that lipids are not only highly effective tumor promotors but are also able to induce resistance against anticancer therapy. Duong et al. provided a list of 12 references that document, based on cell culture and animal experiments, the resistance-inducing effects of adipocytes. The affected therapies range from classical chemotherapies with cisplatin over radiation to antibody treatment with trastuzumab^89^. In an animal experiment, Iwamoto et al. demonstrated that resistance to antiangiogenic therapy (AAT) is lipid-driven and depends on the composition of the hosting tissue. An adipose environment mediates resistance, in contrast to non-adipose tissue^96^. It is believed that FAO can help tumor cells within an adipose environment overcome the energy deficiency induced by AAT^97^. Moreover, the adipose environment seems to maintain low-grade inflammation with the production of radical oxygen species (ROS). These species, however, are needed to establish the cell-damaging effect of radiation. Therefore, it can be assumed that this competitive ROS production decreases the radiogenic effect in lipid-driven cancers.

### Lipid metabolism as a potential therapy target

Considering its high potential as a tumor-promoting factor and ability to induce therapy resistance, it seems obvious that attacking lipid metabolism, especially in cancers that can be assumed to be lipid driven is a promising treatment approach. There exists a plethora of different substances that interact at different sites and levels of lipid metabolism. Supplementary Table 1 gives an overview over a selection of lipid metabolism targets and substances that has been tested in cancers.

One major approach is to reduce the supply or availability of lipids. While previous anti-FASN compounds showed problematic side-effect profiles, TVB2640 (denifanstat), a newer substance, entered phase I studies of lung, ovarian, and breast cancers, among others^98^, and a phase II study of high-grade astrocytoma^99^ and showed a favorable safety profile and promising response rates, especially in combination with other anti-cancer drugs. Attacking CD36 is a promising option for cutting off the FA uptake of tumor cells. Instead of CD36 expression inhibitors, more recent studies have used anti-CD36 antibodies and inhibiting small molecules^100^. These have been shown to be effective preclinically in prostate and gastric cancers, among others^101,102^. There is a long list of inhibitors of FABP4, which is another protein that can serve as a target in anti-cancer therapy. BMS309403 (an FABP4 inhibitor) has been developed for the treatment of metabolic syndrome. However, it has been shown to effectively inhibit ovarian cancer tumor growth in cell culture and animal experiments and has an additive effect in combination with chemotherapy^88,103^. The tumor-promoting effect of a high-fat diet was abrogated by this substance in a mouse model of prostate cancer^104^. Several compounds inhibit SREBPs. These include silibin, nelvinavir, and fatostatin, which have been shown in cell culture and animal experiments to have anticancer effects in breast, prostate, and hepatocellular cancers^105–111^. SREBPs can also be inhibited by pathway activation. Since 1982, metformin, an antidiabetic compound that has been available for a long time, has been investigated in thousands of studies of its anticancer potential. Despite these huge efforts and the high number of available meta-analyses, the real anticancer potential of metformin is still unclear due to conflicting results. In the last two years, several meta-analyses have revealed an anticancer effect, especially in esophageal, gastric, colorectal, and pancreatic cancers^112–115^. However, there have been major points of criticism regarding observational studies. Suissa et al. argued that many of these studies were hampered by time-related biases, while prospective studies could not confirm the positive results of experimental and observational studies^116,117^. Only a few phase III studies have been sufficiently powered.

Glycogen-like peptide-1 receptor agonists (GLP1RAs) gained an important role in the treatment of type-2 diabetes and obesity. However, they also have favorable side effects on the function of the cardiovascular system, kidneys, the central nervous system, and cancer^118^. While a cancerogenic effect in thyroids has been suggested^119^ beneficial effects of the GLP1RAs have been reported regarding prostate, lung, and colon cancer^119^. Due to its metabolic approach a preferable effect on lipid-driven cancers is thinkable.

#### Another concept involves attacking the additional metabolism of intracellularly present lipids

Several lipases are required to build up FAs from neutral fat stores. Inhibitors of adipose triglyceride lipase and monoacylglycerol are available that have been successfully tested in colorectal cancer, among others^36,120^. The inhibition of stearoyl-CoA desaturases-1 influences the saturation status of FAs and has been shown to be effective as an anticancer therapy^121^. Carnitine-palmitoyl-transferase-1 (CPT-1) inhibitors have also shown anticancer efficacy by attacking FAO with direct antiproliferative effects and increasing the efficacy of other therapies, such as chemo- or radiotherapy^36^.

#### Synthetic lethality

The concept of synthetic lethality follows a strategy to combine very different therapies to increase the efficacy of these by heightening the vulnerability of tumor cells or avoiding resistance. The above-mentioned combination of CPT-1 inhibitors with chemo- or radiation therapy is an example of this approach. In this context, it seems important that such strategies need a careful analysis of how a tumor is dependent on lipid metabolism and how a certain neoplasm realizes its need for lipids. A lipogenesis-driven tumor will not be responsive to lipid uptake−attacking approaches, such as anti-CD36^36^.

## Relationship between SARIFA and metabolic reprogramming in cancer

As mentioned above, the strong prognostic power of SARIFA has been demonstrated in several cancer types^1,2,7–10,16^. Our quantitative analyses have shown that even a single tiny SARIFA area within a tumor is enough to indicate a high risk of an adverse clinical course, arguing for a certain biology behind it^20^. Histomorphology strongly suggests the mechanistic relevance of the direct contact of tumor cells with fat cells. Tumor-associated adipocytes shrink (Fig. 1), which is very likely a consequence of the release of fatty acids into the environment, particularly into tumor cells, which uptake these lipids (Fig. 2c and d - lipid staining in SARIFA-positive tumor cells) with the help of FABP4 and or CD36. The morphology in SARIFA-positive cases is identical to that in animal experiments, elucidating the role of lipid metabolism in cancer^88,122,123^. The results of bulk and spatial RNA-expression analyses in SARIFA-positive cases are striking, highlighting the role of upregulated lipid metabolism in concert with the activation of tissue proteases. In particular, the very pronounced increase of FABP4 at the RNA and protein levels in both tumor cells and tumor stroma is a strong indicator of the validity of the hypothesis that the prognostic effect of SARIFA is driven by metabolic reprogramming^1,7,18^. As described in the previous section, numerous studies have demonstrated that FABP4 can increase the aggressiveness of cancers, including their ability to metastasize. It is worth noting that the upregulation of FABP4 is highly influenced by direct interactions between tumor cells and adipocytes. If FABP4 is experimentally downregulated on a genetic or pharmacological level, tumor progression is reduced^78–85^. Tumor cells are able to induce lipolysis in adipocytes by several factors (e.g. catecholamines and cytokines)^92^. The transfer of FAs from the adipocytes to the tumor cells is realized by CD36 and FABP4. The function as an energy source by FAO is only one mechanism that gives a tremendous advantage. Astonishingly, this advantage is maintained when those cells enter the circulation and implant at distanced sites^47,90^. We believe that such lipid metabolism-derived mechanisms are indeed responsible for the aggressiveness of SARIFA-positive cancer, which we could demonstrate. Also, our first indications regarding an impaired immune response in SARIFA-positive cancers fit a lipid-metabolism-enforced carcinogenesis^7,29^. Once this concept finds acceptance, a large amount of evidence from basic research in the field of cancer-related lipid metabolism can be applied to SARIFA-positive cancers. Importantly, SARIFA classification identifies lipid-driven cases within certain tumor entities. Therefore, future lipid metabolism–attacking therapy studies need to stratify between “lipid-hot” and “lipid-cold” tumors. SARIFA seems to be a perfect biomarker for this.

## Limitations and future directions

A huge body of evidence has documented the enormous potential of lipids to promote cancer. Basic research regarding lipid metabolism in the context of cancer has often been performed, assuming that obesity is a causal or enforcing condition. Moreover, several cancer entities have been identified as preferentially promoted by lipids. SARIFA, as a new biomarker, has been shown to be highly prognostic in different cancers. Moreover, SARIFA-positive tumors clearly show the features of lipid-driven cancers. However, our results indicate that SARIFA is not necessarily associated with obesity. Moreover, within a certain cancer entity, there is heterogeneity regarding the occurrence of SARIFAs. It, therefore, seems reasonable not to apply the results regarding lipid metabolism from basic research to all types of cancer or entire entities but to follow a personalized approach. We strongly believe that the SARIFA classification can discriminate between lipid-driven (lipid hot) and nonlipid-driven (lipid cold) cancers. Based on this classification, lipid metabolism–targeting therapies could be stratified.

A limitation of this review is the fact that up to now, SARIFA, except for a very recently published study^19^, has only been investigated by the group of the authors of this review article. Despite all efforts to be objective, a personally biased view cannot be ruled, entirely. It is also important to emphasize that the part regarding the basic research has a narrative nature and is not based on systematic literature research.

A major limitation of the SARIFA biomarker itself is the need for a surgical specimen to evaluate SARIFA status. Therefore, finding surrogate markers that can substitute for the histopathological evaluation of the tumor invasion front seems to be of utmost importance. By correlating SARIFA-status and stromal morphometry^18^, we could already show that SARIFA-positive CRCs are characterized by a significantly lower proportion of tumor (PoT), as defined by West et al.^124^, and also a lower number of vessels (each *p* <0.01) - both measured at the luminal endoscopically reachable tumor surface^18^. These morphologic differences suggest that SARIFA status may be assessable at biopsy specimens in the future if further studies build upon this. The TAF approach of Wulczyn et al. also underlines the power of digital pathology that potentially could help to transfer our concepts to biopsies. We have already proven that SARIFA-positive CRCs also show histological differences in their luminal, endoscopically reachable tumor components, which may serve as a proxy for SARIFA classification in biopsies if further studies build upon these findings. Appropriate serum or plasma markers or expression profiles of tissue biopsies from the tumor surface might be feasible approaches^125^. The mechanisms yielding a neoplastic process with markedly reduced stromal reactions are currently not fully understood. Previous studies have indicated that typical driver mutations do not play a role, in contrast to the immune^7,8^ and wound healing systems^27^. In this context, a possible influence on the efficacy of immunotherapy should be addressed. Therefore, further investigations should be performed in this direction. Moreover, next to the cancer entities already investigated, numerous other epithelial cancers, such as urothelial cancer, squamous cell cancers of various localizations, and breast and ovarian cancers, as well as melanomas, sarcomas, and hematologic neoplasms, should be evaluated regarding the potential effects of SARIFA. Interestingly, currently, unpublished preliminary results from investigations regarding breast cancer indicate a correlation between the molecular type and SARIFA-positivity. The latter seems to occur predominantly in hormone-driven luminal-A cancers and might be associated with a favorable course. Large-scale investigations will be necessary to elucidate this contradictory phenomenon. In general, it seems likely that lipid-driven tumors follow a certain metastatic route that prefers lipid-rich metastatic niches such as bones or the omental site. The first preliminary data of our own investigation already point in this direction. As outlined above, attacking lipid metabolism is an attractive concept. SARIFA could serve as an effective biomarker for selecting those cases with the highest potential to benefit from such regimes. Newer drugs like GLP1RA should be evaluated at an experimental level while other existing substances can be considered where broad experimental evidence can be considered sufficient for phase-I and II trials.

### Supplementary information

Supplementary Table and Fugures

## Data Availability

No datasets were generated or analyzed for this article. This word does not include novel data but only refers to original contributions.
