# Supplementary material for: The SARIFA biomarker in the context of basic research of lipid-driven cancers
Source: NPJ Precis Oncol. 2024 Jul 31;8:165. doi: 10.1038/s41698-024-00662-2 (PMC11291993; doi:10.1038/s41698-024-00662-2)
Supplement: Supplementary file 1 — Supplementary Table and Fugures [file 41698_2024_662_MOESM1_ESM.pdf]

The SARIFA biomarker in the context of basic research of lipid-driven cancers

Bruno Märkl[1,3,4*], Nic G. Reitsam[1,3,4*], Przemyslaw Grochowski[1,3,4], Johanna Waidhauser[,2,3,4], Bianca Grosser[1,3,4]

* These authors contributed equally.

[1]Pathology, Medical Faculty Augsburg, University of Augsburg, Germany
[2]Hematology and Oncology, Augsburg, University of Augsburg, Germany
[3]Bavarian Cancer Research Center (BZKF), Augsburg, Germany
[4]WERA Comprehensive Cancer Center, Augsburg, Germany

Supplementary Table 1 – Potential substances for targeting lipid metabolism focused on the targets FABP, CD36 and CPT1; mod. acc. to (Butler, Perone et al. 2020) and (Röhrig and Schulze 2016).

Target	Agent	Preclinical model or clinical trial	Comment	Reference
**FABP**	EI-05	Preclinical	Inhibition of tumor growth of mammary tumor in mouse model	(Rao, Singh et al. 2015)
	SBFI-102, 103	Preclinical	Increase in cytotoxic and tumor-suppressive effects of taxanes in Prostate cancer cells.	(Carbonetti, Converso et al. 2020)
	BMS309403	Preclinical	Impairment of growth and metastases of tumor in several mouse models	(Uehara, Takahashi et al. 2014, Nie, Zhang et al. 2017, Laouirem, Sannier et al. 2019, Wang, Yao et al. 2019, Mukherjee, Chiang et al. 2020, Tian, Zhang et al. 2020, Yang, Deng et al. 2021, Sun and Zhao 2022)
**CD36**	ABT-510	Phase II	ABT-510 therapy in patients with metastatic melanoma did not demonstrate definite clinical efficacy	(Markovic, Suman et al. 2007)
	ABT-510	Preclinical	Inhibition of tumor growth and ascites formation in a preclinical model of human ovarian cancer	(Greenaway, Henkin et al. 2009)
	VT1021	Phase II/III	Activation of CD36-mediated apoptotic signaling in cancer cells (Breast<br>Glioblastoma, Ovarian, Pancreatic cancers)	(Mahalingam, Harb et al. 2020); NCT03364400
**CPT1**	Etomoxir	Preclinical/Clinical trials stopped due to toxicity	Suppression of tumor progression in bladder cancer cell line; decreased prostate cancer tumor growth in mouse model;<br>Inhibition of proliferation of leukaemia and myeloma cell lines	(Cheng, Wang et al. 2019); (Schlaepfer, Rider et al. 2014); (Samudio, Harmancey et al. 2010); (Tirado-Vélez, Joumady et al. 2012)
	Perhexiline	Preclinical/Approved New Zealand (Angina pectoris)	Reduction in tumor growth of primary leukaemia cells and TCL1 transgenic mice	(Liu, Liu et al. 2016)
	ST1326	Preclinical	Growth inhibition and apoptosis of prostate cancer cells	(Brusselmans, De Schrijver et al. 2005)

Supplementary References:

Brusselmans, K., E. De Schrijver, G. Verhoeven and J. V. Swinnen (2005). "RNA interference–mediated silencing of the acetyl-CoA-carboxylase-α gene induces growth inhibition and apoptosis of prostate cancer cells." Cancer research **65**(15): 6719-6725.

Butler, L. M., Y. Perone, J. Dehairs, L. E. Lupien, V. de Laat, A. Talebi, M. Loda, W. B. Kinlaw and J. V. Swinnen (2020). "Lipids and cancer: Emerging roles in pathogenesis, diagnosis and therapeutic intervention." Adv Drug Deliv Rev **159**: 245-293.

Carbonetti, G., C. Converso, T. Clement, C. Wang, L. C. Trotman, I. Ojima and M. Kaczocha (2020). "Docetaxel/cabazitaxel and fatty acid binding protein 5 inhibitors produce synergistic inhibition of prostate cancer growth." Prostate **80**(1): 88-98.

Cheng, S., G. Wang, Y. Wang, L. Cai, K. Qian, L. Ju, X. Liu, Y. Xiao and X. Wang (2019). "Fatty acid oxidation inhibitor etomoxir suppresses tumor progression and induces cell cycle arrest via PPARγ-mediated pathway in bladder cancer." Clin Sci (Lond) **133**(15): 1745-1758.

Greenaway, J., J. Henkin, J. Lawler, R. Moorehead and J. Petrik (2009). "ABT-510 induces tumor cell apoptosis and inhibits ovarian tumor growth in an orthotopic, syngeneic model of epithelial ovarian cancer." Mol Cancer Ther **8**(1): 64-74.

Laouirem, S., A. Sannier, E. Norkowski, F. Cauchy, S. Doblas, P. E. Rautou, M. Albuquerque, P. Garteiser, L. Sognigbé and J. Raffenne (2019). "Endothelial fatty liver binding protein 4: a new targetable mediator in hepatocellular carcinoma related to metabolic syndrome." Oncogene **38**(16): 3033-3046.

Liu, P. P., J. Liu, W. Q. Jiang, J. S. Carew, M. A. Ogasawara, H. Pelicano, C. M. Croce, Z. Estrov, R. H. Xu, M. J. Keating and P. Huang (2016). "Elimination of chronic lymphocytic leukemia cells in stromal microenvironment by targeting CPT with an antiangina drug perhexiline." Oncogene **35**(43): 5663-5673.

Mahalingam, D., W. Harb, A. Patnaik, S. Ulahannan, H. Mahdi, M. Ahluwalia, M. Patel, A. Dowlati, A. Bullock, P. Wen, S. Pant, M. Mulcahy, R. Guttendorf, L. Vaickus, S. Wang, M. Crochiere, M. Vincent, M. Cieslewicz and J. Watnick (2020). "374 A first-in-human Phase 1/2 open label trial evaluating the safety, pharmacology, and preliminary efficacy of VT1021 in subjects with advanced solid tumors." Journal for ImmunoTherapy of Cancer **8**(Suppl 3): A228-A228.

Markovic, S. N., V. J. Suman, R. A. Rao, J. N. Ingle, J. S. Kaur, L. A. Erickson, H. C. Pitot, G. A. Croghan, R. R. McWilliams, J. Merchan, L. A. Kottschade, W. K. Nevala, C. B. Uhl, J. Allred and E. T. Creagan (2007). "A phase II study of ABT-510 (thrombospondin-1 analog) for the treatment of metastatic melanoma." Am J Clin Oncol **30**(3): 303-309.

Mukherjee, A., C. Y. Chiang, H. A. Daifotis, K. M. Nieman, J. F. Fahrmann, R. R. Lastra, I. L. Romero, O. Fiehn and E. Lengyel (2020). "Adipocyte-Induced FABP4 Expression in Ovarian Cancer Cells Promotes Metastasis and Mediates Carboplatin Resistance." Cancer Res **80**(8): 1748-1761.

Nie, J., J. Zhang, L. Wang, L. Lu, Q. Yuan, F. An, S. Zhang and Y. Jiao (2017). "Adipocytes promote cholangiocarcinoma metastasis through fatty acid binding protein 4." J Exp Clin Cancer Res **36**(1): 183.

Rao, E., P. Singh, X. Zhai, Y. Li, G. Zhu, Y. Zhang, J. Hao, Y. I. Chi, R. E. Brown, M. P. Cleary and B. Li (2015). "Inhibition of tumor growth by a newly-identified activator for epidermal fatty acid binding protein." Oncotarget **6**(10): 7815-7827.

Röhrig, F. and A. Schulze (2016). "The multifaceted roles of fatty acid synthesis in cancer." Nature Reviews Cancer **16**(11): 732-749.

Samudio, I., R. Harmancey, M. Fiegl, H. Kantarjian, M. Konopleva, B. Korchin, K. Kaluarachchi, W. Bornmann, S. Duvvuri, H. Taegtmeyer and M. Andreeff (2010). "Pharmacologic inhibition of fatty acid oxidation sensitizes human leukemia cells to apoptosis induction." J Clin Invest **120**(1): 142-156.

Schlaepfer, I. R., L. Rider, L. U. Rodrigues, M. A. Gijón, C. T. Pac, L. Romero, A. Cimic, S. J. Sirintrapun, L. M. Glodé, R. H. Eckel and S. D. Cramer (2014). "Lipid catabolism via CPT1 as a therapeutic target for prostate cancer." Mol Cancer Ther **13**(10): 2361-2371.

Sun, N. and X. Zhao (2022). "Therapeutic Implications of FABP4 in Cancer: An Emerging Target to Tackle Cancer." Front Pharmacol **13**: 948610.

Tian, W., W. Zhang, Y. Zhang, T. Zhu, Y. Hua, H. Li, Q. Zhang and M. Xia (2020). "FABP4 promotes invasion and metastasis of colon cancer by regulating fatty acid transport." Cancer Cell Int **20**: 512.

Tirado-Vélez, J. M., I. Joumady, A. Sáez-Benito, I. Cózar-Castellano and G. Perdomo (2012). "Inhibition of fatty acid metabolism reduces human myeloma cells proliferation." PLoS One **7**(9): e46484.

Uehara, H., T. Takahashi, M. Oha, H. Ogawa and K. Izumi (2014). "Exogenous fatty acid binding protein 4 promotes human prostate cancer cell progression." Int J Cancer **135**(11): 2558-2568.

Wang, S., Y. Yao, X. Wang, G. Zheng, W. Ouyang and W. Chen (2019). "25-HC promotes hepatocellular carcinoma metastasis through up-regulation of TLR4 dependent FABP4." Am J Cancer Res **9**(10): 2140-2155.

Yang, H., Q. Deng, T. Ni, Y. Liu, L. Lu, H. Dai, H. Wang and W. Yang (2021). "Targeted Inhibition of LPL/FABP4/CPT1 fatty acid metabolic axis can effectively prevent the progression of nonalcoholic steatohepatitis to liver cancer." Int J Biol Sci **17**(15): 4207-4222.

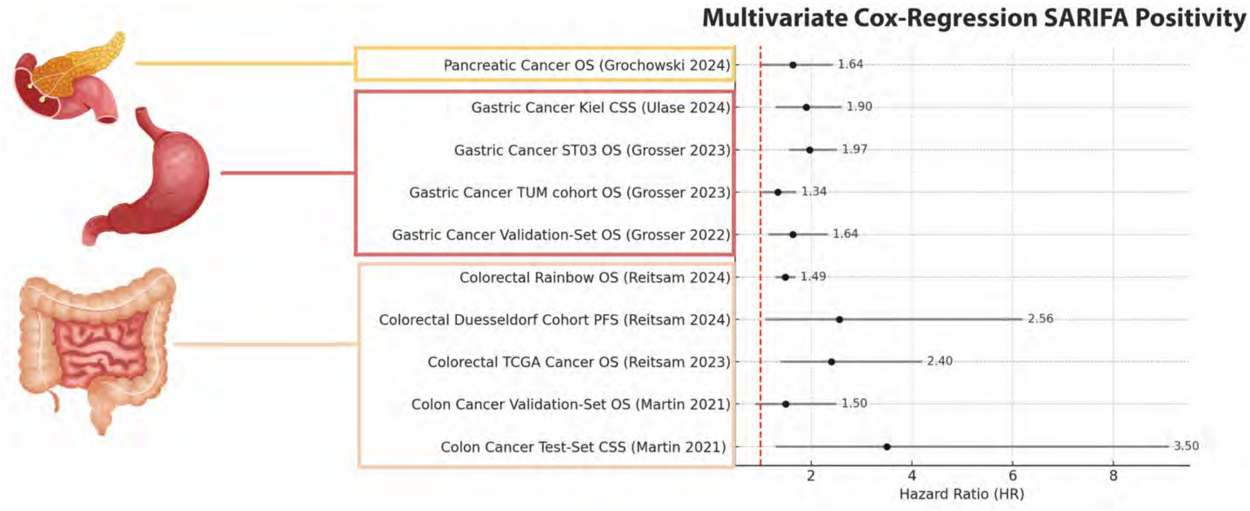

Supplementary Figure 1: Multivariate Cox-Regression Analyses for different tumor entities. OS = overall survival, CSS = cancer-specific survival, PFS = progression-free survival

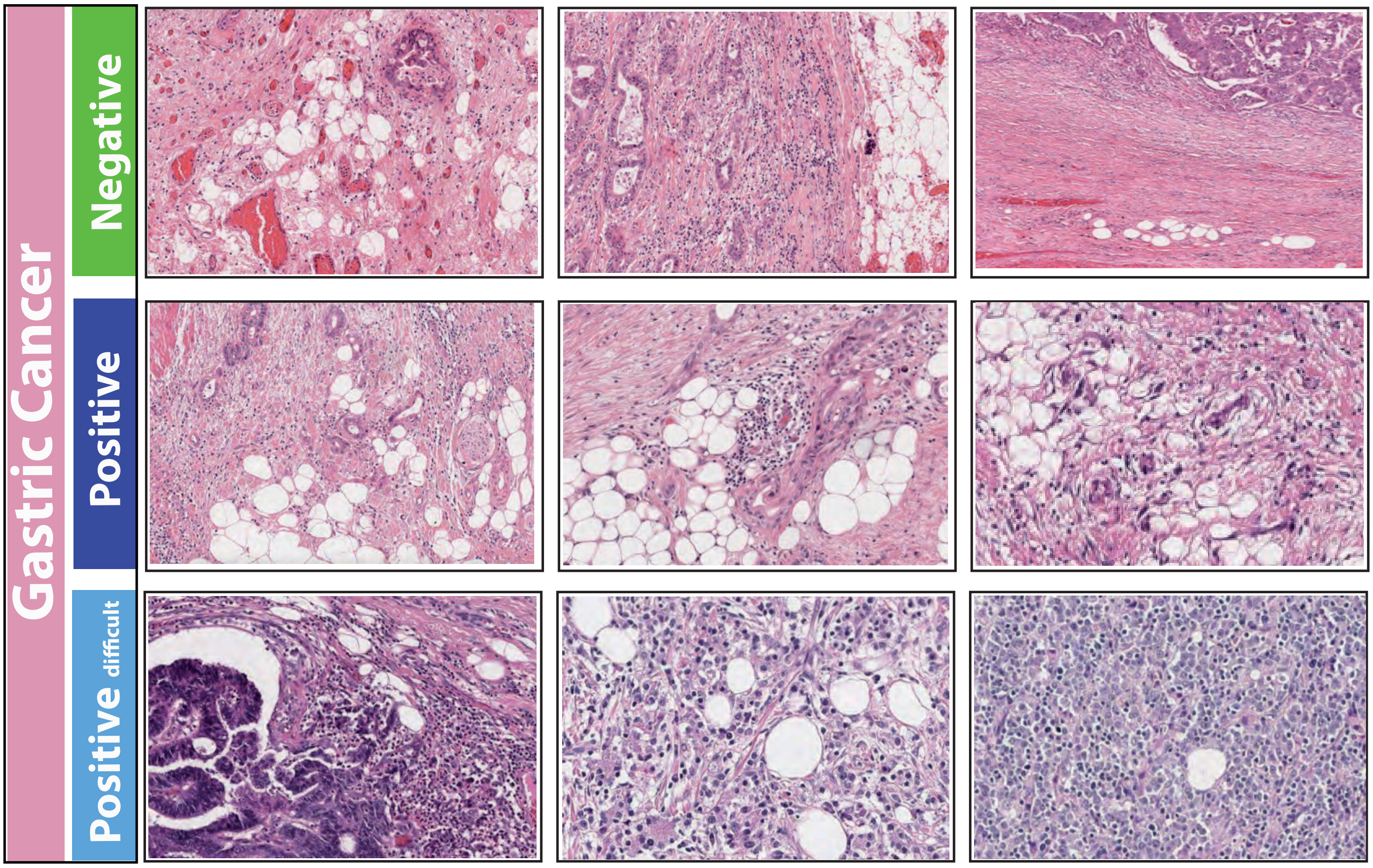

Supplementary Figure 2: H&E, Gastric cancer cases. Upper row: SARIFA-negative examples; middle row: SARIFA-positive examples; lower row: SARIFA-positive examples with suptile morphology.

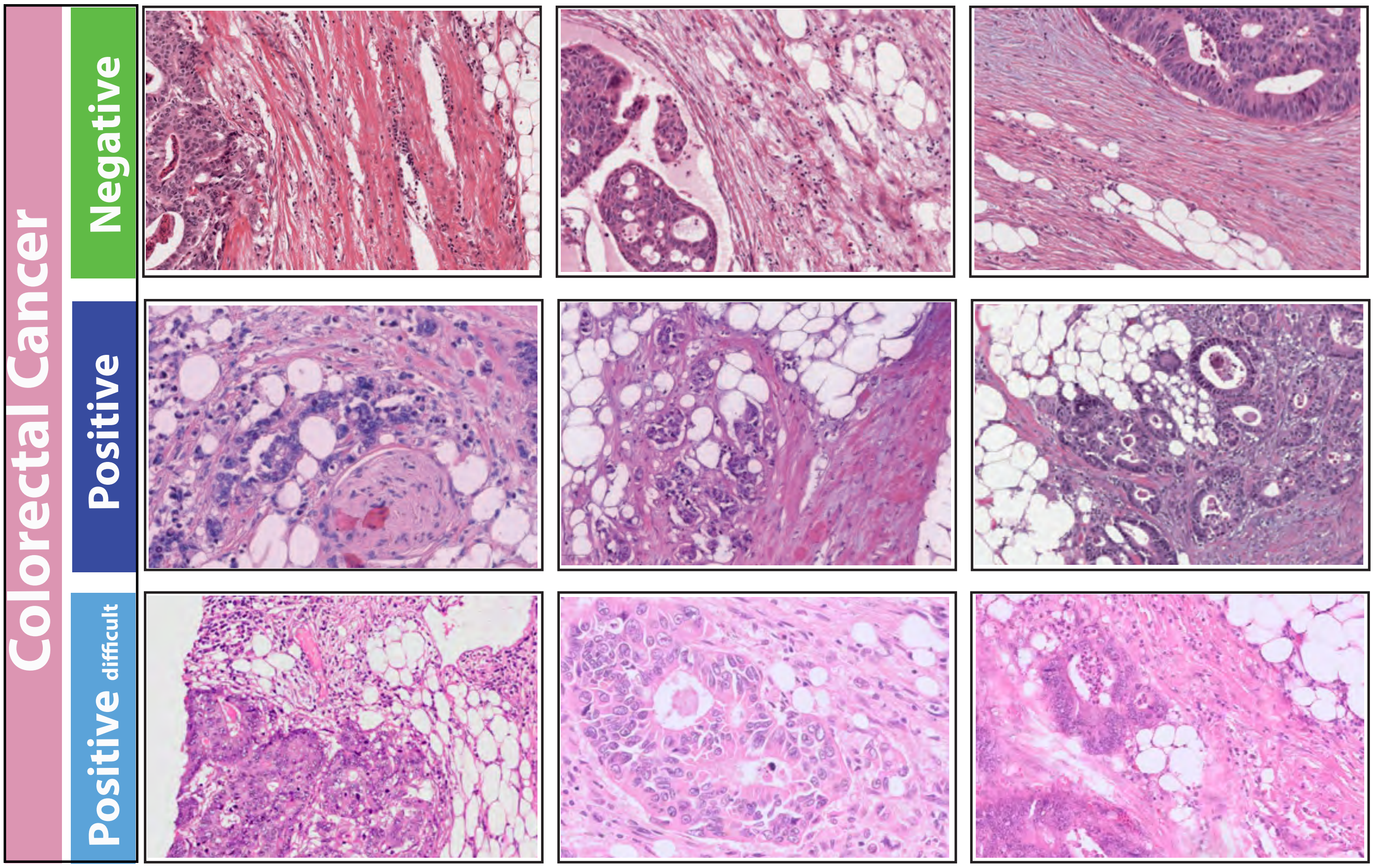

Supplemantary Figure 3: H&E, Colorectal cancer cases. Upper row: SARIFA-negative examples; middle row: SARIFA-positive examples; lower row: SARIFA-positive examples with suptile morphology.

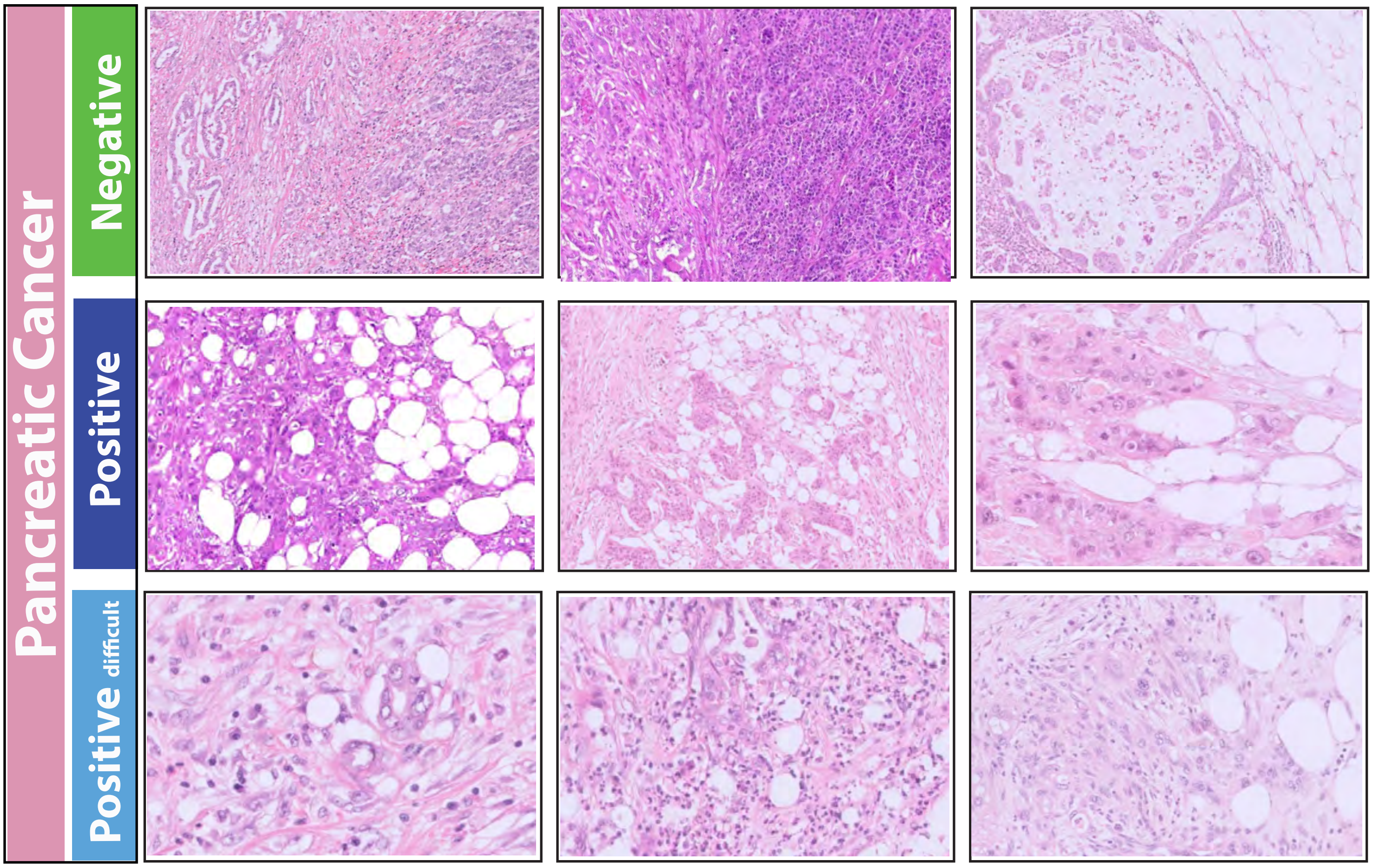

Supplementary Figure 4: H&E, Pancreatic cancer cases. Upper row: SARIFA-negative examples; middle row: SARIFA-positive examples; lower row: SARIFA-positive examples with suptile morphology.

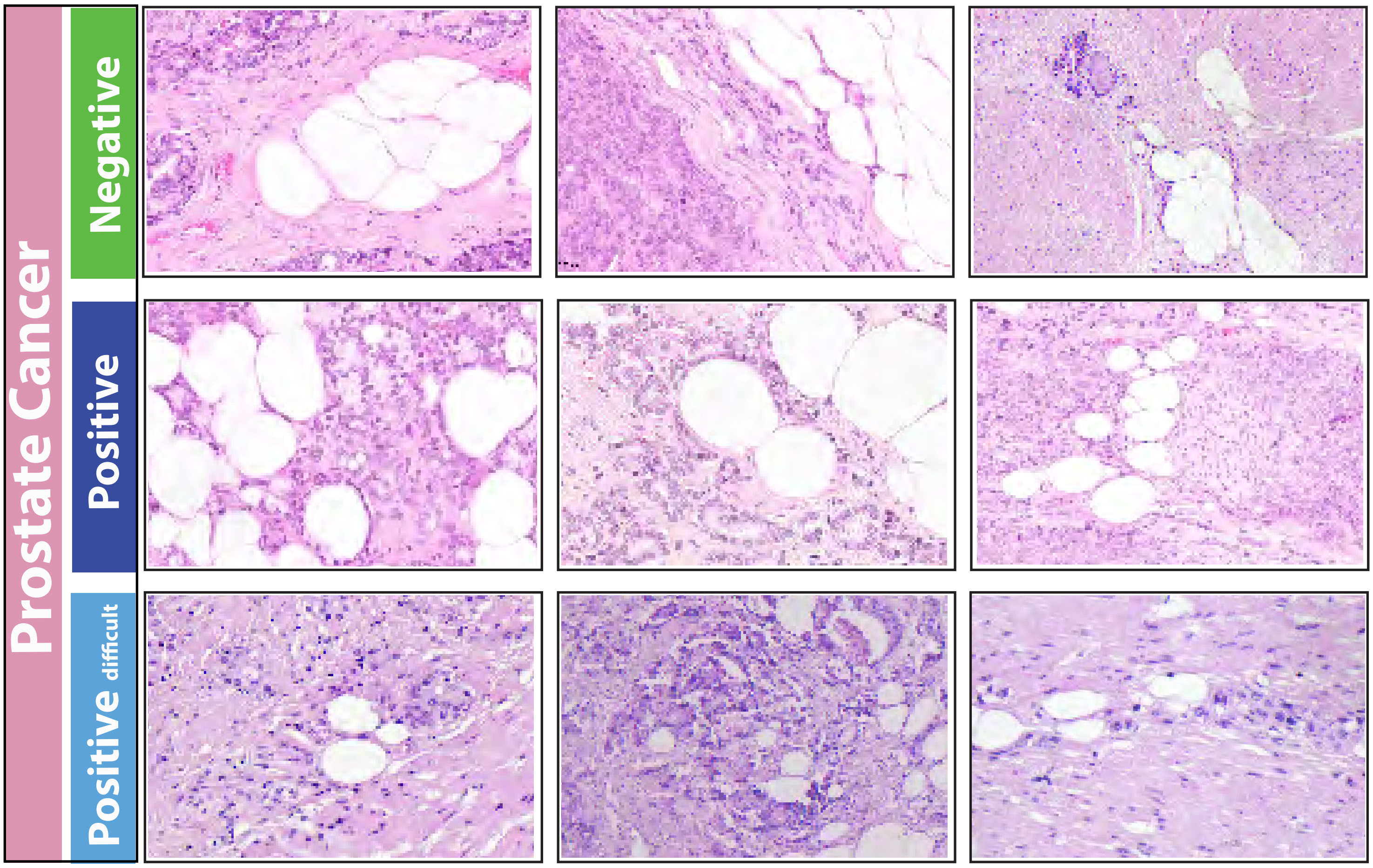

Supplementary Figure 5: H&E, Prostate cancer cases. Upper row: SARIFA-negative examples; middle row: SARIFA-positive examples; lower row: SARIFA-positive examples with suptile morphology.
